# Bactericidal activity of some plant essential oils against *Ralstonia solanacearum* infection

**DOI:** 10.1016/j.sjbs.2021.11.045

**Published:** 2021-11-24

**Authors:** Rahma Abd-Elrahim, Mohamed R.A. Tohamy, Mahmoud M. Atia, Mohamed M.A. Elashtokhy, Mohamed A.S. Ali

**Affiliations:** a Plant Pathology Dept., Faculty of Agriculture, Zagazig University, Zagazig 44511, Egypt; b Genetics Dept., Faculty of Agriculture, Zagazig University, Zagazig 44511, Egypt

**Keywords:** *Ralstonia solanacearum*, Antibacterial activity, Essential plant oils, Ezymes, Transmission electron microscope (TEM)

## Abstract

Potato plants and their tubers in Egypt are affected by one of the most renowned soil-borne pathogen, *Ralstonia solanacearum*, that caused brown rot in potato tubers and wilt in plants. There is no efficient therapeutic bactericide so; control of bacterial wilt is very rough.

The study investigated three different concentrations of seven essential plant oils under *in vitro* and *in vivo* conditions as a result of their effects on *Ralstonia solanacearum* growth and their possibility use as potato seed pieces dressing for controlling bacterial wilt disease incidence. *In vitro*, anise oil at the three tested different concentrations (0.04, 0.07, and 0.14% vol/vol) was the most effective one inhibiting the growth of T4 and W9 isolates of *Ralstonia solanacearum* then pursued by thyme, lemongrass, and clove oils. On the other hand, rocket oil at the tested concentration was the least effective one followed by fennel oil. However, wheat germ oil was not completely effective. *In vivo,* experiment revealed that anise oil at the three concentrations significantly reduced disease incidence and severity in sponta and hermes potato cultivars and their effect was associated with increase of peroxidase, polyphenoloxidase, phenols and the foliar fresh weight of treated plants as well as the weight of tubers/plant followed by thyme and lemongrass oils compared to the infected untreated control.

Morphological differences in bacterial cell structure have been observed using a transmission electron microscope (TEM). Anise oil at higher concentration caused of cell wall rupture and degraded cellular components.

## 1 Introduction

Bacterial wilt (BW) symptoms in fields of potato plants known as 'brown rot symptoms' in potato tubers during storage is caused by *Pseudomonas solanacearum *(Smith 1896) Yabuuchi et al. currently in 1995 called *Ralstonia solanacearum*. It is a seed and soil-borne pathogen and has a wide host range worldwide (Salanoubat et al. 2002).

Control of bacterial wilt is complicated due to the existence of many pathogenic strains and its wide host range (Hayward, 1991, Mahbou Somo Toukam et al., 2009). Controlling this disease through resistant cultivars is ineffective since resistance in the host plant may be variable according to that bacterium which is seed and soil-borne pathogen and the different host environmental growth conditions (Lopez and Biosca 2004).

Medicinal essential plant oils and their active components are renowned as having fungicidal and bactericide effects (Ji et al. 2005). Precursory *in vitro* and *in vivo* experiments were conducted using several essential plant oils and their components showed that some leaf thyme has significant efficacy against *R. solanacearum* (Pradhanang et al. 2003). The use of essential plant oils is a potential pathway to control brown rot of potato tubers disease as reported by Oboo et al. (2014). He inferred these oils have antibacterial activity that is effective in the control of *R. solanacearum* that caused brown rot in potato tubers.

Plant extracts have antimicrobial effects on plant pathogens. Besides plants, essential plant oils include several volatile compounds, such as aliphatic aldehydes, terpenoids, esters and alcohols (Dewick 1997). Also, are used as biofumigants in an integrated disease management system (Ji et al. 2007).

Different plant compounds are elected to control *R. solanacearum* in the field. The antimicrobial effects of the essential plant oils on the pathogens such as *R. solanacearum* have been scrutinized worldwide (Deberdt et al., 2012, Lucas et al., 2012, Moghaddam et al., 2014).

Thyme essential oil is rich in thymol and carvacrol. So, it could be suitable in controlling plant diseases (Lee et al., 2005, Uysal et al., 2015) and also their derivatives being affluent in phenolic acids (Roby et al., 2013, Erturk et al., 2017).

Plant disease resistance is correlated with the activation of defense means that slacken infection at certain stages of the host-pathogen interaction. Various defense enzymes including Peroxidase (PO) and polyphenoloxidase (PPO) are serious in the early reaction of the host to the infection, (Adss 2013). Cumulative incorporation of phenolic compounds within the cell wall during incompatible plant–microbe/elicitor interactions often lead to increase PO activity (Soares et al. 2005).

Peroxidase and polyphenoloxidase are the most defense-related enzymes in plants against pathogens, (Qin et al. 2015). Polyphenoloxidase (PPO) and peroxidase (PO) are distinctive antioxidant enzymes and remarkable components of defense against membrane lipid peroxidation and oxidative stress during pathogen infestation (Foyer and Noctor 2003).

From the above, the study aimed to achieve several axes, namely (i) Evaluates the antibacterial effects of seven essential plant oils on *R. solanacearum* growth *in vitro*. (ii) Controlling *R. solanacearum in vivo* to reduce incidence and severity of potato wilt by improving an efficient application methods and finally (iii) Evaluation the effect of essential plant oils on the vegetative growth of ptotato plants, the activity of enzymes and phenols in treated potato plants.

## 2 Materials and methods

### 2.1 Bacterial culture and inoculum preparation

Two bacterial isolates (T4 and W9) were previously isolated from potato tubers and water, identified and used in this study, (Abd-Elrahim et al. 2015).

A well-characterized *R. solanacearum* (T4 isolate) race 3 biotype 2 by the Quantitative, Real-time, Fluorogenic PCR (*Taq*-Man) assay was previously conducted in Potato Brown Rot Project (PBRP), Agric. Res. Center, Giza –Egypt (Abd-Elrahim et al. 2015).

The two bacterial pathogenic isolates (T4 and W9) were grown at 28 °C either on Tetrazolium chloride medium (TZC) agar for 48 h. Colonies of tested bacterial isolates, based on their colony morphology, were harvested and suspended in liquid culture of casamino acids, peptone, and glucose (CPG) and grown at 28 °C for three days (Kelman 1954). Cultures were centrifuged at 10,000 rpm for 10 min at 10 °C. Bacterial pellets were suspended in distilled water and adjusted to 10^8^cfu /ml.

### 2.2 Plant essential oils

Highly purified essential plant oils *i.e.* Anise (*Pimpinella anisum* L), Thyme (*Thymus vulgaris* L), Clove (*Syzigium aromaticum* L), Fennel (*Foeniculum vulgare* L), Germ wheat (*Triticum vulgare* L), Rocket (*Eruca sativa* L) and lemongrass (*Cymbopogon flexuosus* L) were kindly obtained from National Research Center, Giza, Egypt and used for the current study. Three concentrations were prepared from each original oil (0.04, 0.07, and 0.14% vol/vol) and tested based on results of a former study (Pradhanang et al. 2003) on *R. solanacearum* strain.

The tested essential plant oils were emulsified in Tween 20 (1:1 ratio) to improve their solubility in water. Twenty milliliters of water were amended with 8 μl of the oil to give a final concentration of 0.04% vol/vol. Similarly, 14 and 28 μl of the oils were emulsified and added to 20 ml of water for final concentrations of 0.07 and 0.14% vol/vol, respectively.

### 2.3 *In vitro* effect of essential plant oils on *Ralstonia solanacearum* growth

The experiments were carried out at Microbial Genetics Research laboratory, Genentics Dept., Fac. Agric., Zag. Univ., Zagazig, Egypt.

The antagonistic effect of essential plant oils against the growth of *R. solanacearum* (T4 and W9) was studied on King^'^s B agar plates (King et al. 1954). One hundred microliters of *R. solanacearum* suspension containing 10^8^cfu/ml was suspended in soft agar and spread on the plate surface using a sterilized L glass shape rod spreader. Then a 7 mm diameter agar disk (cut from a plate on which the essential oils) was placed on its center. The inhibition zone around the disc was measured as mm after 48 hrs.

### 2.4 *In vivo* treatment application

This study was conducted in greenhouse of Plant Path Dept., Fac. Agric., Zag. Univ., Zagazig, Egypt.

Uniform tubers of two cultivars (sponta and hermes) were kindly provided by PBRP. Potato seed pieces were planted in plastic sterilized pots (25 cm) filled with 10Kg *Ralstonia solanacearum* free sandy-clay soil (1/1, v/v) obtained from pest-free fields (zagazig) with one tuber/ pot. Soil infestation was carried out with *R. solanacearum* isolate suspensions (10^8^ cfu) (T4), previously propagated in casamino acids, peptone, and glucose (CPG) liquid culture and grown for three days at 28 °C by adding 50 ml/kg soil. Only five essential oils *i.e.* anise, thyme, clove, fennel, and lemongrass proved to be effective *in vitro* experiment, were used at three tested concentrations (0.04, 0.07, and 0.14% vol/vol). Tubers were soaked separately in 250 ml of each essential plant oil concentration for 15 min so that the buds are not damaged then planted in pots contain infested soil. Control treatment pots were maintained under the same conditions without infestation and only treated with concentrations of tested essential plant oils. Five pots were used for each particular treatment.

Disease incidence was calculated as the percentage of wilt plants after 45 days of planting.

Disease severity was determined 45 days after the first sign of disease incidence according to the following modified scale suggested by Winstead and Kelman (1952):

The disease severity was calculated according to the following equation:

$$Diseaseseverity\%=\frac{\sum (No.ofwiltedplantsineachcategory\times wiltgrade)}{TotalNo.ofplants\times highestgrade}\times 100$$

Reduction Percent of disease incidence and severity were calculated using the following formula**:**

$$PercentReduction=\frac{C-T}{C}X100$$

C = control T = treatment

Weight of tubers (g) and fresh weight of plant (g/plant) were calculated after 90 days after planting, while enzyme activity and phenol contents were calculated after 45 days of planting.

### 2.5 Enzymes activity

Enzymes were determined from fresh samples of potato leaves (1 g) collected from the infected plants of 45 days after planting.

#### 2.5.1 Peroxidase activity

Peroxidase was calculated according to Kochba et al. (1977). The enzyme was calculated as color density read in spectrophotometer 601 at 425 nm wavelength and measured as mg/g fresh weight.

#### 2.5.2 Polyphenoloxidase activity

Polyphenoloxidase was calculated according to Lisker et al. (1983). The enzyme was calculated as color density read in spectrophotometer 601 at 495 nm wavelength and measured as mg/g fresh weight.

### 2.6 Total phenol contents

Total phenols were extracted by adding one gram of potato leaf pieces which were collected from the infected plants 45 days after planting to 10 ml ethanol 70% on a water bath at 70 °C for 72 h. and left to dry, then 5 ml isopropanol 50% was added to dry film (residue) and kept in the deep freezer for chemical determinations.

One ml of the previously prepared sample extract was determined according to the method described by Snell and Snell (1953). On an absorbance spectrophotometer 601 at 520 nm wavelength the color optical density of the reacted mixture, was measured.

### 2.7 Transmission electron microscope

Transmission electron microscopy (TEM) has been used to characterize morphology of bacterial cells before and after dealing with the most effective essential plant oil (anise) at 0.14% concentration. The overnight cultured bacterial suspension (isolate T4) was supplemented with anise essential oil at 0.14% concentration and incubated at 30 °C for 24 h. Transmission electron microscopy images were obtained and photographed using a Negative stain -transmission electron microscope (Cairo University) with a working voltage of 80 kV.

### 2.8 Statistical analysis

Data have been statistically analyzed (Gomez and Gomez 1984), and means were compared using L.S.D (Duncan 1955).

## 3 Results

**Fig 1:**
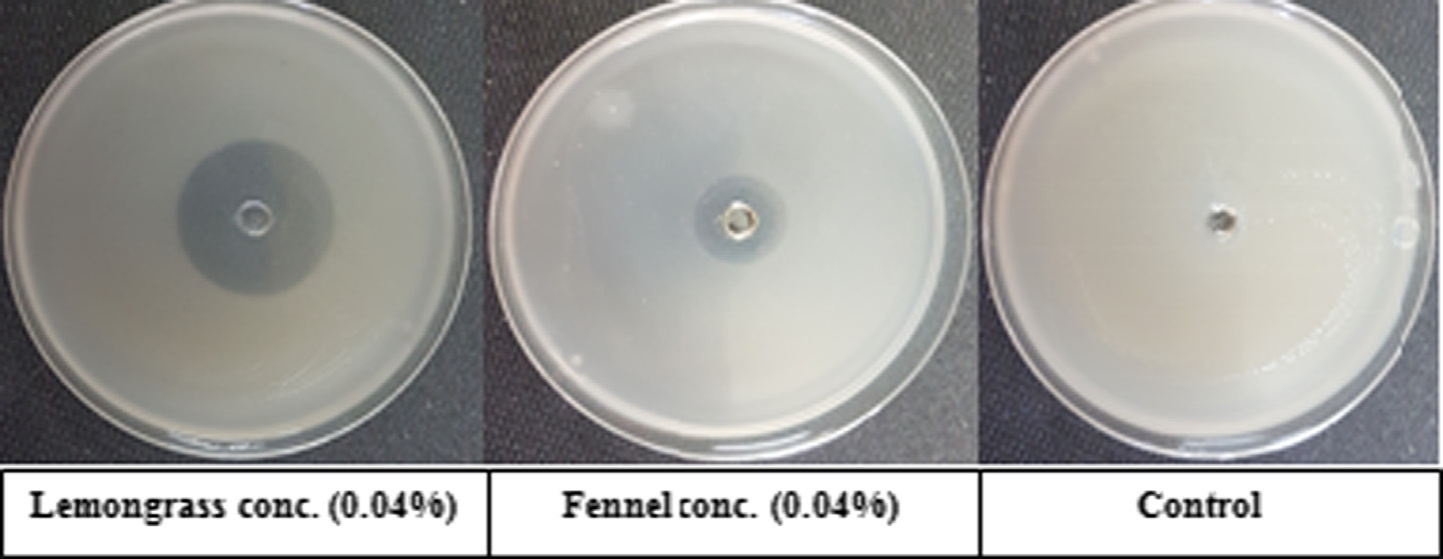
*In vitro* antagonistic activities of lemongrass and fennel at 0.04% essential plant oils against *Ralstonia solanacearum* isolate W9 on King^'^s B medium.

It is clear from data in Table 1 and Fig. 1 that all the used essential plant oils at different concentrations inhibit *Ralstonia solanacearum* growth on the agar plate except for wheat germ oil. The results revealed that anise oil at the three tested concentrations was the most efficient in inhibiting the two *Ralstonia solanacearum* isolates growth followed by thyme, lemongrass and clove oils in the three tested concentrations (0.04, 0.07 and 0.14% v/v). The results on w9 isolate were recorded as 66.7, 60.3, 57; 52, 49, 44; 40.7, 37.7, 34.3; 30.3, 24 and 20.3 mm, respectively. On the other hand, rocket and fennel oils at different concentrations exhibit the least effective ones followed by fennel oil with values 6, 7.7, 10.3; 12, 14.7 and 17.3 mm, respectively. However, wheat germ oil at the three tested concentrations was not effective against the two *Ralstonia solanacearum* isolates.

**Table 1:** *In vitro* antagonistic activities of tested essential plant oils against two *Ralstonia solanacearum* isolates expressed as inhibition zone (mm).

**Essential oil**	**Concentration**	**Isolate**	
		**T4**	**W9**
Anise	0.04%	51	57
	0.07%	56.3	60.3
	0.14%	62.3	66.7
Thyme	0.04%	38.7	44
	0.07%	45.3	49
	0.14%	49	52
Lemongrass	0.04%	26.7	34.3
	0.07%	31	37.7
	0.14%	34	40.7
Clove	0.04%	17.3	20.3
	0.07%	19.3	24
	0.14%	20.7	30.3
Fennel	0.04%	9.6	12
	0.07%	10.7	14.7
	0.14%	14	17.3
Rocket	0.04%	3.7	6
	0.07%	5.7	7.7
	0.14%	7.7	10.3
Wheat germ	0.04%	0	0
	0.07%	0	0
	0.14%	0	0
LSD at 0.05		1.5086	1.3247

**Fig 2:**
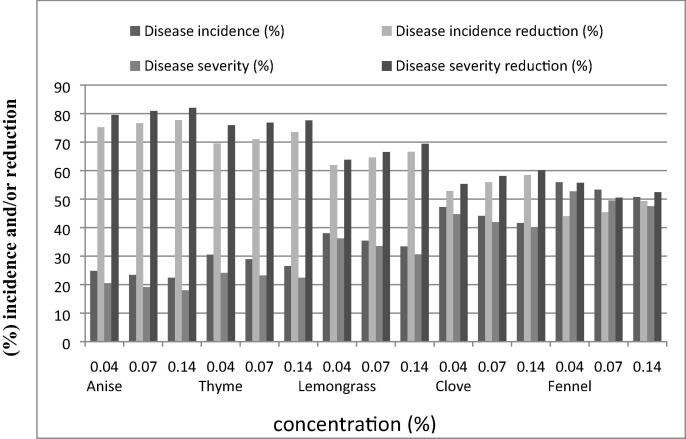
*In vivo* effect of tested essential plant oils on reduction percentage of wilt disease incidence and severity of hermes cultivar infected with *Ralstonia solanacearum* (isolate T4)*.*

**Fig. 3:**
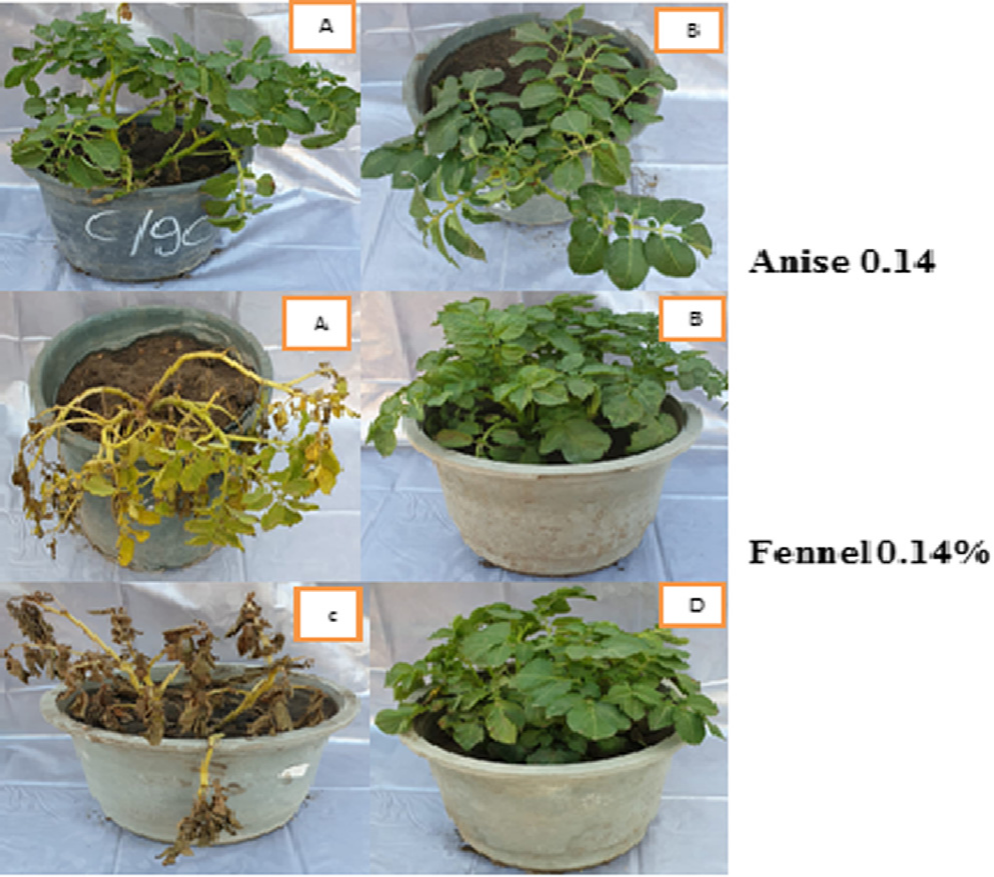
*In vivo* effect of essential oils at 0.14% on potato wilt disease incidence (Hermes cultivar).

Table 2 and Fig 2, Fig. 3 showed the differences in percentage wilt disease incidence and severity reduction caused by the virulence *R. solanacearum* (isolate T4) among the tested essential plant oils on the two potato cultivars (hermes and sponta) under greenhouse conditions. Anise oil was the most effective in reducing potato wilt disease on hermes and sponta cultivars resulting the highest reduction percentage of disease incidence followed by thyme oil and lemongrass, respectively compared to the infested untreated control treatment. On the other hand, fennel oil exhibited the least reduction percentage of both disease incidence and severity while clove revealed moderate values.

**Table 2:** *In vivo* effect of tested essential plant oils on reduction percentage of wilt disease incidence and severity of hermes and sponta cultivars infected with *Ralstonia solanacearum* (isolate T4)*.*

**Essential oil**	**Concentration** **(%)**	**Hermes**		**Sponta**	
		**Incidence reduction (%)**	**Severity reduction (%)**	**Incidence reduction (%)**	**Severity reduction (%)**
Anise	0.04	75.2	79.5	59.6	63.1
	0.07	76.6	80.9	61.7	64.9
	0.14	77.7	82	64.2	67.4
Thyme	0.04	69.5	75.9	53.8	54.9
	0.07	71	76.8	56.5	58.4
	0.14	73.5	77.6	58	60.7
Lemongrass	0.04	61.9	63.8	43.5	46.9
	0.07	64.6	66.5	45.4	48.9
	0.14	66.6	69.4	49	51.5
Clove	0.04	52.8	55.3	36	37.8
	0.07	55.9	58.1	38.5	41.3
	0.14	58.4	60.1	40.5	43.9
Fennel	0.04	44	55.7	23.7	27.5
	0.07	45.4	50.5	27.8	31.2
	0.14	49.3	52.4	31.8	34.4
Infected untreated		00.0	00.0	00.0	00.0
Healthy control		100.0	100.0	100.0	100.0
LSD at 0.05		1.0074	0.8872	4.6130	0.8971

**Fig 4:**
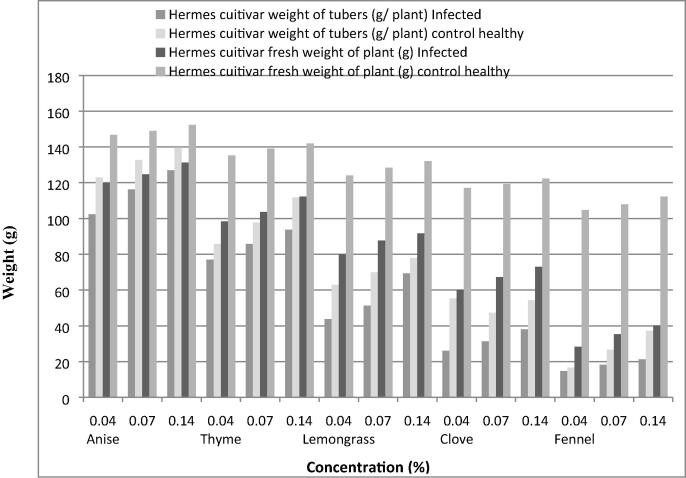
*In vivo* effect of tested essential plant oils on some plant growth parameters of wilted potato plants (Hermes cultivar) infected with *Ralstonia solanacearum* (T4).

**Fig 5:**
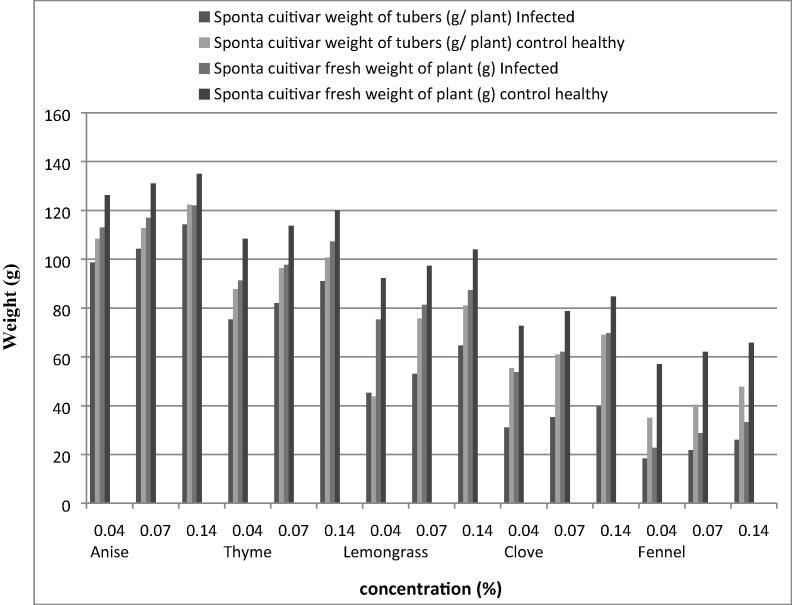
*In vivo* effect of tested essential plant oils on some plant growth parameters of wilted potato plants (Sponta cultivar) infected with *Ralstonia solanacearum* (T4).

Data presented in Fig 4, Fig 5 reveal that anise, thyme and lemongrass oils at the three tested concentrations increased average fresh weight and tubers/plant of hermes and sponta cultivars, respectively. On the other hand, fennel oil and clove oil exhibit the least average weight of tubers/plant and fresh weight of plant, respectively.

**Fig 6:**
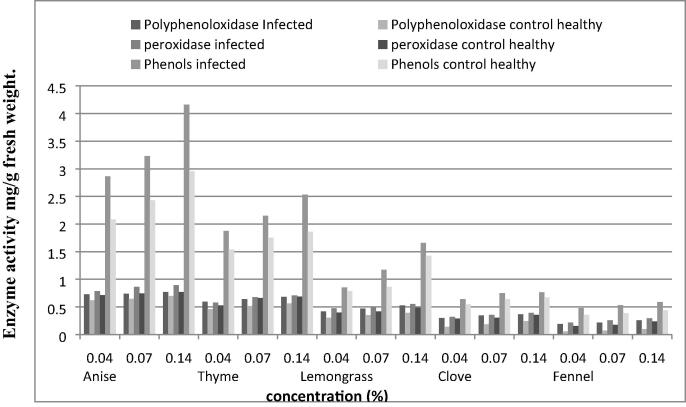
The activity of peroxidase, polyphenoloxidase and total phenol contents measured as mg/g fresh weight of sponta potato plant leaves cultivar treated with essential plant oils in *Ralstonia solanacearum* (T4) infected plants.

Data in Fig. 6 showed that the highest activity values of peroxidase, polyphenoloxidase and total phenol contents of sponta cultivar was calculated with anise, thyme and lemongrass at 0.14, 0.07 and 0.04% concentrations calculating 0.895, 0.863 , 0.786; 0.708, 0.677, 0.579; 0.551, 0.497and 0.475 for peroxidase, 0.769, 0.741, 0.727; 0.684, 0.641, 0.595; 0.528, 0.472 and 0.416 for polyphenoloxidase and 4.163, 3.231, 2.864; 2.531, 2.148, 1.874; 1.658, 1.174 and 0.853 for total phenol contents, respectively.

While the lowest activity values 0.217, 0.256, 0.295; 0.318, 0.358 and 0.391 for peroxidase, 0.191, 0.217, 0.256; 0.302, 0.347 and 0.368 for polyphenoloxidase and 0.482, 0.532, 0.587; 0.642, 0.748 and 0.765 for total phenol contents, respectively of fennel and clove.

**Fig 7:**
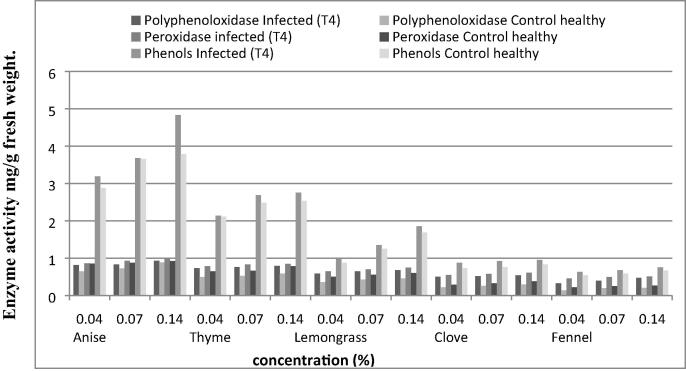
The activity of peroxidase, polyphenoloxidase and total phenol contents measured as mg/g fresh weight of Hermes potato plant leaves cultivar treated with essential plant oils of *Ralstonia solanacearum* (T4) infected plants.

Data in Fig. 7 showed that the highest activity values of peroxidase, polyphenol oxidase and total phenol contents of hermes cultivar was calculated with anise while thyme and lemongrass revealed moderate values at 0.14, 0.07 and 0.04% concentrations being 0.985, 0.927, 0.856; 0.846, 0.828, 0.785; 0.742, 0.698 and 0.647 for peroxidase, 0.932, 0.832, 0.816; 0.792, 0.758, 0.595; 0.675, 0.648 and 0.584 for polyphenoloxidase 4.832, 3.682, 3.186; 2.753, 2.685, 2.139; 1.853, 1.349 and 0.982 for total phenol contents, respectively.

The least activity values being 0.451, 0.496, 0.507; 0.543, 0.578 and 0.607 for peroxidase, 0.326, 0.397, 0.468; 0.504, 0.514 and 0.538 for polyphenoloxidase, 0.628, 0.674, 0.751; 0.872, 0.921 and 0.953 for total phenol contents were of fennel and clove.

TEM was used to check the interaction between the anise essential plant oil and the bacterial cells (isolate T4) to compare the cellular variations of the untreated and treated cells during the treatment with anise essential oil.

**Fig 8:**
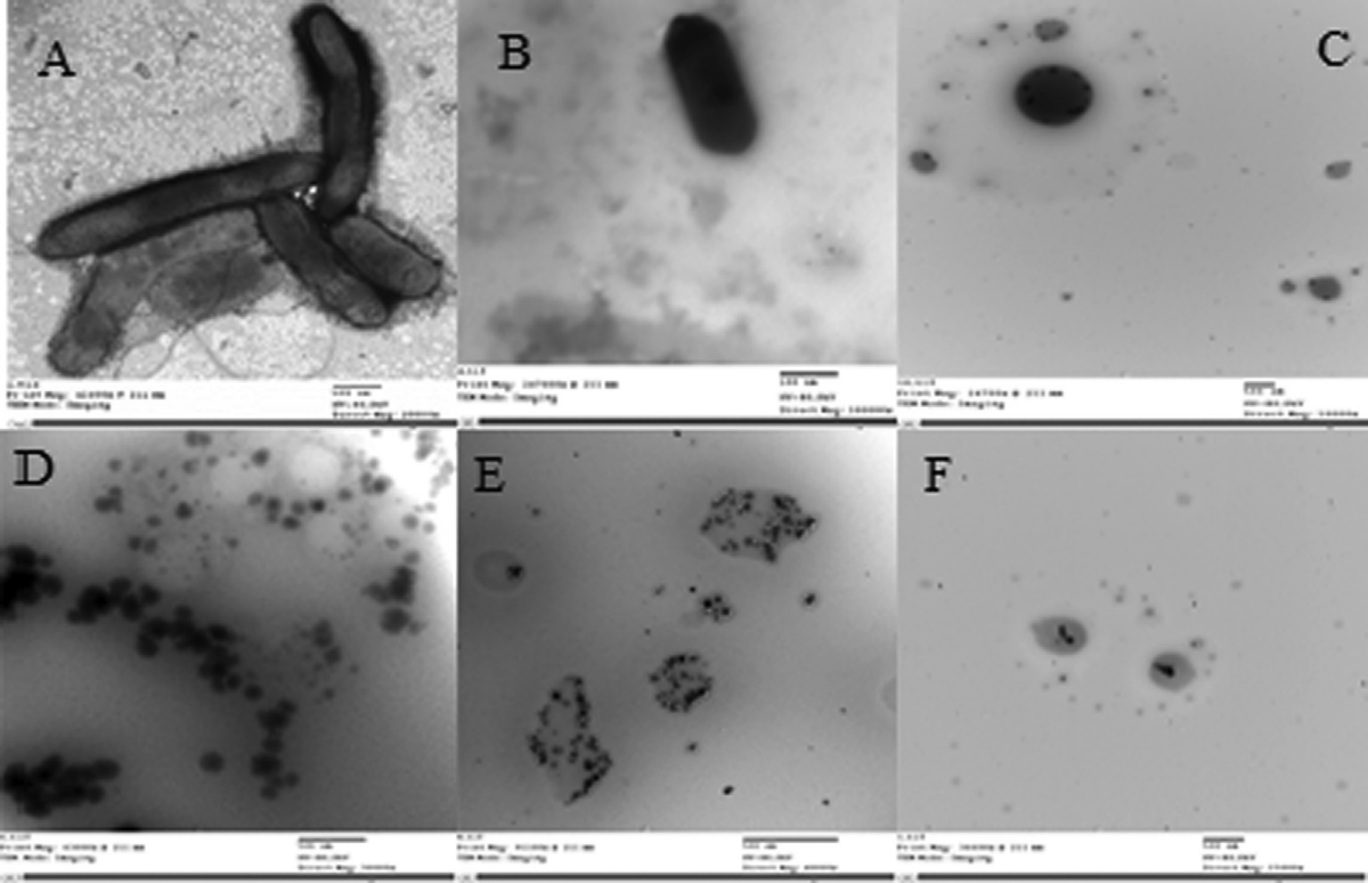
TEM images of (A, B) *R. solanacearum* cells untreated with Anise essential oil. (C, D, E, F) The overnight cultured bacterial suspension (isolate T4) was supplemented with anise essential oil at 0.14% concentration and incubated at 30 °C for 24 h. Observations of the damaged bacterial membranes of the anise essential oil-treated bacteria using TEM (C). Cells treated with anise oil were twisted in shape (D) bubble- like structures were noticed outside the cell and ruptured. Large masses of debris were attacked to the cells (E) the cells did not have a smooth texture and were smaller in size compared with untreated controls and (F) The cytoplasm of damaged cells was dense.

As evident in TEM micrographs (Fig. 8), untreated *R. solanacearum* cells with tested essential oils were soft texture. Cells treated with anise oil were wrapped up in form and bleb-like structures outside the cell which lacerated. Large masses of debris were collected. Also, the cells have a tortuous texture and were smaller in size compared with untreated controls.

## 4 Discussion

The absence of the most effective chemical control treatments for sanitizing *Ralstonia solanacearum* infected fields led to manage *R. solanacearum* that caused bacterial wilt disease of potato is currently highly rough. The most reliable strategy to control *R. solanacearum* is through the genetic program to introduce resistant cultivars (Lebeau et al. 2011) but this strategy is unavailable in Egypt due to that all potato seed pieces are imported. The present study was carried out to evaluate the efficiency of seven available essential plant oils (anise, thyme, fennel, germ wheat, clove, rocket, and lemongrass) *in vitro* against the growth of two pathogenic *R. solanacearum* isolates (T4 and W9) and the possibilities of using these plant oils *in vivo* to control potato bacterial wilt.

These results demonstrated that anise, thyme, lemongrass, and fennel essential plant oils capable to suppress growth of *R. solanacearum in vitro* (isolate T4 and W9) and their effect relying on the concentration used.

The tested five essential plant oils were under *in vitro* were selected according to their results as they are the most efficient inhibiting of *R. solanacearum* growth. It is worthy to mention that wheat germ oil at different concentrations used was not efficient in inhibiting the growth of the two *R. solanacearum* isolates. Also, the rocket has low affected inhibiting effect on the growth of *R. solanacearum*. Therefore, wheat germ oil and rocket oil were excluded from *in vivo* study.

Our results showed that anise, thyme, lemongrass, and fennel essential plant oils reduced growth of *R. solanacearum* (isolate T4) *in vivo* at different concentration.

The obtained results concerning the reduction effects of bacterial wilt *in vivo* are in agreement with former reports obtained by (Pradhanang et al. (2003). They found that oils of palmarosa and lemongrass reduced populations of *R. solanacearum* race- 1 and the bacterial wilt of tomato. Also, Paret et al., 2010, Hong et al., 2011 demonstrated the bactericidal effect of palmarosa, thymol, and lemongrass on *R. solanacearum* race 4, which cause bacterial wilt of tomato and edible ginger in tropical areas. Also, Huang and Lakshman (2010) explicated that clove oil has the prospect to eliminate populations of *R. solanacearum* in soil and reduce the incidence of geranium wilts and tomato bacterial wilt *in vivo* conditions. Similar results have been found on the antimicrobial effects of thyme oil against *R. solanacearum* and *Pectobacterium carotovorum. (RF.)* Nezhad et al. (2012).

Expound the mode of action of essential plant oils on inhibiting the growth of pathogenic bacteria and disease control depended on classifying the contents of these essential oils and their effects on pathogens growth and their roles in plants metabolism after application.

In this respect, several research works were carried out to explain the mode of action of such essential plant oils depended on the oil, pathogen, host plant, and the interaction between them. Among these researchs were recorded by Rota et al., 2008, Hindumathy, 2011 They found that the inhibition impact of essential oils on *R. solanacearum* growth can be attached to its active antibacterial compounds that include: steroids, terpenoids, alkaloids, geraniol, citral, flavonoids, eugenol, cytronolal, geranyl acetate, beta cariofiln, tannins, phenolic compounds (thymol), terpene hydrocarbons (γ-terpinene) saponin, farnsul, and p-Cymene, respectively.

The present study indicated the highest activity of anise oil against *R. solanacearum.* In this respect, the antimicrobial activities of anise extracts may be attributed to their phenolic contents reported by Martins et al., 2016, Bettaieb Rebey et al., 2018 such as Anethole, Acid linoléque, Limonenene, and Fenchone since numerous phytochemical studies indicated the presence of noticeable amounts of phenolic compounds in anise.

In the case of lemongrass Hindumathy (2011) showed that its inhibiting effect might be due to having alkaloids and phenols such as α-terpinene, linalool, α-pinene, and γ-terpinene contains antibacterial properties.

The antimicrobial properties of the essential oils of thyme were found to be depending mostly on their phenolic constituents. The quantitatively most important compounds are the phenols thymol and carvacrol. It is known that these two phenolic compounds have strong antimicrobial properties (Sivropoulou et al., 1996, Aligiannis et al., 2001). Thyme is also characterized by a high content of monoterpenes, such as hydrocarbons γ-terpinene and β-cymene.

The bacterial activity of fennel was explained according to Anwar et al. (2009) who found that *trans*-anethole, fenchone, estragole, and limonene were the major antibacterial ingredients of the fennel seeds essential oil tested.

Extracted clove oil essential one that contains eugenol which was found to be the main constituent of clove oil, revealed by the chemical structure analyses (Chaieb et al. 2007). A previous study carried out by Walsh et al. (2003) showed that active eugenol, one of the main compounds in clove oil, had antibacterial activity where it has ability to inhibit the function of the membrane of microorganisms such as *E*. *coli*, including the certain processes or cellular enzymes.

Also, mechanisms of antibacterial activity of essential plant oils were explained by Ultee et al., 1999, Dorman and Deans, 2000, Rota et al., 2008. They found that clove essential oil can harms several essential bacterial cell processes, such as the change in DNA synthesis, the wastage of turgidity, the inhibition of enzymes activity and the decreasing of metabolic processes through the hydrophobic constituent of carvacrol, eugenol, eugenol acetate, and caryophyllene present in clove essential oil reacts with the cell membranes of the alter bacteria, alteration the permeability for H + and K + cations.

The action mechanisms of essential plant oils were found also to be included in the disintegration of the cell wall (Helander et al., 1998, Gill and Holley, 2006), destroying cytoplasm coagulation specially cytoplasmic membrane (Gustafson et al. 1998), destroying the membrane proteins, increased permeability leading to leakage of the cell contents (Lambert et al. 2001).

On the other hand, essential oils also alter membrane permeability by destroying the electron transport system (Tassou et al. 2000) and several components of the essential oils such as carvon, thymol, and carvacrol, lead to an increase in the intracellular concentration of ATP (Helander et al. 1998). Inhibiting electron transport, protein translocation, synthesis of cellular components. Finally all physiological changes that can result in cell lysis and death (Ben Arfa et al. 2006).

Essential oils consist of low-molecular-weight, oxygen analogs, and phenol derivatives (Adminis et al., 2002, Huang et al., 2005). The small sizes of essential oil molecules easily penetrate through cell walls and affect diverse biochemical processes.

Oils with a phenolic content, such as thymol, have a high antimicrobial activity against bacteria, as explained in many studies (Misharina and Samusenko, 2008, Bassolé et al., 2010, Soković et al., 2010). Several suppositions have been proposed to demonstrate this mode of action of essential plant oils. Kallali et al. (2020) found that total phenolic content was significantly raised in inoculated and non-inoculated tomato plants with bacteria by fennel seed essential oil.

The present results showed that treated potato plants with essential plant oils (anise, thyme and lemongrass) increased phenols in inoculated potato plants. In addition, results obtained are identical to those as stated by Benhamou et al. (2000). This report proposed that phenolic compounds may prevent pathogen infection by increasing the mechanical force of host cell walls which lead to the inhibition of pathogen infection. Flavonoids are the polyphenolic compounds formed by plants for reducing oxidative stress in cells (Panche et al. 2016). Also, results showed that the raised content of phenol in potato plants inoculated with pathogen and treated with the essential oil may be due to increasing systemic resistance in the host plants (Rajeswari 2014). The constituent defenses of plants include structural hurdles, such as plant cell walls as well as inhibitory compounds including phenolic compounds (Nürnberger et al. 2004). These phenolic compounds might play serious role in the plant's resistance to pathogens. It is considered one of the most antimicrobial defense compounds (Dixon et al. 2002).

Ultee et al. (1999) found that the hydrophobic component of carvacrol in the clove oil interacts with the cell membranes of bacteria and decreased the permeability for H + and K + cations. This alteration harms essential cell processes, such as enzymes by the loss of those ions, the loss of turgidity, the alteration in DNA synthesis, and the reduction of metabolic activities.

Some of the plant extracts are commonly rich in phenolic compounds (phenolic acids and flavonoids) and tannins that had antioxidant and antimicrobial properties. Phenolic compounds are hydrogen donors and are able to remove free radicals and reduce oxidative harm (Kesharwani et al., 2012, Wintola and Afolayan, 2015), which makes it a powerful antioxidant. In addition to the antioxidant activity, phenolic compounds act as antimicrobial agents via several mechanisms including the lysis of microbial membranes (Proestos et al., 2006, Bajpai et al., 2009).

Under greenhouse conditions, yield components showed a proportional relationship with the efficacy of the essential oils. These results are concord with those previously reported by other researchers using plant essential oils to control bacterial wilt of tomato *in vivo*. Pradhanang et al., 2003, Ji et al., 2005, Lee et al., 2012 reported that thyme oil treatment significantly decreased bacterial wilt incidence of tomato and increased vegetative growth compared to the untreated control.

Obtained results demonstrated that anise, thyme and lemongrass essential plant oils can increase activity values of peroxidase and polyphenoloxidase. However, fennel essential oil reveals the least ones. The role of enzymes in plants treated with oils was recorded by many research workers, among them Vanitha and Umesha (2008) who indicated that the protection enzymes lipoxygenase (LOX) and polyphenol-oxidase (POX) are actively participated in the resistance to bacterial wilt incidence. It may inhibit the growth of *Ralstonia solanacearum*.

Increased POX activity has been found in the resistant reactions in plant- bacterial interactions (Young et al. 1995). The increase in activities of POX is associated with the rate of pathogen multiplication and spread suggesting an active role for POX in plant resistance (Reimers et al., 1992, Guo et al., 1993). The enhancement of POX activity was confirmed during several host-pathogen interaction systems (Kartashova et al. 2000).

In response to pathogen invasion, the host produces defense enzymes, which are involved in resistance to pathogens. The defense enzymes play serious role in plants defense plants against pathogens. Plants have their specific defense enzymes as PPO, PO and-1, 3-glucanase. Peroxidase enzymes that are an answerable for both scavenging of H_2_O_2_ by the oxidation of phenols (Polle et al. 1994).

Peroxidase is an important enzyme during defense reaction against pathogen in the oxidation of phenols and lignification of host plant cells. Polyphenoloxidase is important in the oxidation of polyphenols into quinones and lignification of plant cells during microbial infection (Mohammadi and Kazemi 2002).

The microscopic images of treated and untreated plant cells observed by TEM displayed the treated cells with anise oil had a lysis semblance, showing a direct effect of anise essential oil on cell structure of pathogen. The cell membrane was penetrated, a further significance of the antibacterial properties of anise essential oil. The cytoplasm of pathogen treated cells was bushy and black whereas untreated cells showed the perfect negative stain around the cell membrane but not within the cell (Santiago et al 2019).

## Consent to Participate Publish

All authors Consent to Participate and publish.

## Availability of data and materials

Not applicable.

## Ethical Approval

Not applicable.

## Funding

Authors were funding this research work themselves as well as authors analysis, and interpretation of data and in writing the manuscript.

## Declaration of Competing Interest

The authors declare that they have no known competing financial interests or personal relationships that could have appeared to influence the work reported in this paper.
